# The Association of Readiness for Interprofessional Learning with empathy, motivation and professional identity development in medical students

**DOI:** 10.1186/s12909-018-1248-5

**Published:** 2018-06-07

**Authors:** Cora L. F. Visser, Janneke A. Wilschut, Ulviye Isik, Stéphanie M. E. van der Burgt, Gerda Croiset, Rashmi A. Kusurkar

**Affiliations:** 1 VUmc School of Medical Sciences & VUmc Amstel Academy, VU University Medical Center, Postbus 7057, 1007 MB Amsterdam, the Netherlands; 2 Department of Epidemiology & Biostatistics, Decision Modeling Center, VU University Medical Center, Amsterdam, the Netherlands; 3 VUmc School of Medical Sciences, VU University Medical Center, Amsterdam, The Netherlands; 4 LEARN! Research Institute for Learning and Education, Faculty of Psychology and Education, VU University Amsterdam, Amsterdam, the Netherlands; 5 Present Address: UMC Groningen, Groningen, the Netherlands

**Keywords:** Readiness for IPL, Professional identity development, Empathy, Interprofessional learning, Shared learning

## Abstract

**Background:**

The Readiness for Interprofessional Learning Scale is among the first scales developed for measurement of attitude towards interprofessional learning (IPL). However, the conceptual framework of the RIPLS still lacks clarity. We investigated the association of the RIPLS with professional identity, empathy and motivation, with the intention of relating RIPLS to other well-known concepts in healthcare education, in an attempt to clarify the concept of readiness.

**Methods:**

Readiness for interprofessional learning, professional identity development, empathy and motivation of students for medical school, were measured in all 6 years of the medical curriculum. The association of professional identity development, empathy and motivation with readiness was analyzed using linear regression.

**Results:**

Empathy and motivation significantly explained the variance in RIPLS subscale Teamwork & Collaboration. Gender and belonging to the first study year had a unique positive contribution in explaining the variance of the RIPLS subscales Positive and Negative Professional Identity, whereas motivation had no contribution. More compassionate care, as an affective component of empathy, seemed to diminish readiness for IPL. Professional Identity, measured as affirmation or denial of the identification with a professional group, had no contribution in the explanation of the variance in readiness.

**Conclusions:**

The RIPLS is a suboptimal instrument, which does not clarify the ‘what’ and ‘how’ of IPL in a curriculum. This study suggests that students’ readiness for IPE may benefit from a combination with the cognitive component of empathy (‘Perspective taking’) and elements in the curriculum that promote autonomous motivation.

## Background

Since healthcare relies on teamwork [1–3], medical schools intend to prepare future doctors to collaborate with other healthcare professionals and with lay persons [4]. Interprofessional learning (IPL) was introduced to have students from different healthcare professions learn with, from and about each other, in order to improve interprofessional collaboration (IPC) (caipe.org.uk/resources/defining-ipe). Unfortunately, it has been difficult to engage medical students in IPL, since they focus on the development of a *mono-*professional identity, instead of an interprofessional one [5–9]. Engaging students in IPE and thus influencing their attitudes towards IPC is important, because negative attitudes can stand in the way of learning. The concept of “readiness for interprofessional learning”, which is the measurement of a student’s attitude toward IPL, may suggest how ready health professions education students are for interprofessional learning. The current study investigates the relationships between readiness for IPE, professional identity, empathy and motivation of undergraduate medical students in an attempt to unravel the concept of readiness for IPL.

### Readiness for Interprofessional Learning

In 1999, Parsell and Bligh introduced the concept of ‘Readiness for Interprofessional Learning’ as the degree to which students are willing to participate in IPL, using 4 dimensions: knowledge and skills for teamwork, roles and responsibilities of self and others, benefits to patients, practice and personal growth, and values [10]. Since then, the Readiness for Interprofessional Learning Scale (RIPLS) has been widely used as a pre and post-intervention test of attitudes toward IPL, e.g. [11, 12]. Some studies have used a 4 factor version instead of the original 3 factor version of the RIPLS [13, 14] resulting in a better internal consistency of the subscales, an extended version [15] where the decline in attitudes towards IPL and patient-centeredness suggested that an IPE experience can be ill-embedded in the curriculum, or in combination with focus groups [16] in order to better understand the perceptions of the students regarding IPE and address concerns, e.g. regarding role blurring, in the IPE activity. The RIPLS has been criticized for weaknesses in the reliability of measurements by the subscales [17, 18]. Thus, measurement of the willingness and ability of students for IPL needs further attention. For doing this, it is important to first understand what is readiness for learning in general. In the literature, readiness for learning has mostly been studied in children. It is perceived as a threshold which needs to be passed before learning can occur. According to Tyler (1964) the concept ‘Readiness for learning’ concerns the question ‘When to teach?’ [19]. However, since learning also takes place without formal instruction, is domain-specific and dependent on the way learners have organized knowledge, Watson(1998) advocates asking ‘how to teach’ and ‘what to teach’ [20]. In a study concerning adolescent learners, Conley and French (2014) introduced ‘College readiness’. During college and with specific guidance, students can grow in college readiness i.e. in ‘how to learn’. In the transition from high school to college, the authors consider ownership of learning to be the key component of ‘college readiness’. Ownership of learning consists of 5 components: motivation and engagement, goal orientation and self-direction, self-efficacy and self-confidence, meta-cognition and self-monitoring and persistence [21].

### Professional identity

In the concept ‘readiness for IPL’ the professional identity is included in the dimension ‘benefits to patients, practice and personal growth’ as well as ‘values’. Students need to develop a professional identity, i.e. ‘ways of being and relating in professional contexts’ [22]. Professions are distinguished from one another by the expert knowledge bases they use, expressed in qualifications and competencies. The formation of professional identity is described as “development of professional values, moral principles, actions and aspirations - ultimately a complex structure that an individual uses to link motivations and competencies to a chosen career role” [23]. We consider this complex structure to be the perspective of a profession. Different professions develop different perspectives on situations e.g. patient problems [24, 25]. We hypothesize that medical students who have a stronger professional identity, exhibit lower readiness for IPL. When a patient is being cared for by different professionals, shared values, a shared goal and an attuned care plan are important for ensuring patient safety and good quality of care [26, 27]. McNair (2005) argues that teamwork requires blurring of the boundaries between knowledge and skills of different experts, citing the literature that some professionals perceive this as a threat, undermining IPC. Interprofessionalism should be considered as a framework to include values shared by all healthcare professions [28].

### Empathy

Empathy is one value that healthcare professionals share. Empathy has been defined as having a cognitive component as well as an affective component. The cognitive component refers to taking the perspective of the patient into account during counselling [29]. The affective component refers to the ability to feel warmth, compassion and concern for the patient [30] and ‘to walk in the patient shoes’ [31]. To some extent, healthcare professionals also need these capabilities when working with colleagues from a different profession. IPC is enhanced when healthcare professions bring empathy in their mutual interactions in order to align their policies and achieve the best quality of communication with the patients [32]. Jordan and Foster (2016) conceptualized empathy in physician-patient interactions using Interpersonal Theory, which assumes that social behaviour consists of two dimensions: affiliation (varying from friendly and warm to hostile and cold) and control (varying from dominant and directive to submissive and yielding) [33]. Jordan et al. expected that a person who is warm and friendly in his/her interactions (affiliation), will over time encounter more advantageous social interactions. For the present study, we hypothesize that students who are willing to take the perspective of the patient and are able to feel warmth and concern for the patient, show this capacity with students from other professions in shared learning as well, meaning that higher scores on the empathy subscale ‘taking the perspective of the patient’ are associated with higher scores on readiness for IPL.

Learning to apply the affective component of empathy has benefits for the medical student: Prosocial behaviour increases students’ well-being and vitality even without contact with the recipient [34]. A positive experience can impact the motivation for IPL and IPC.

### Motivation

Motivation has two dimensions: valuing a cause and dedicating time and energy. According to Self-determination theory, the quality of motivation is dynamic and can shift along a continuum ranging from amotivation through controlled motivation to autonomous motivation [35]. Students can be controlled motivated, when their behaviour is guided by (expected) rewards or punishment from others, or they can be more autonomously motivated when their behaviour is guided by their interest or personal endorsement of the activity. We hypothesize that autonomous motivated medical students are more willing to learn interprofessionally. Autonomous motivation is the desirable quality of motivation, as it is associated with deep study strategy and better academic performance in medical students [36].

Professional identity, empathy and motivation have been separately studied in medical students [30, 37–39]. Furthermore, these concepts have been investigated in a number of combinations, e.g. readiness for IPL in combination with professional identity [11] and the combination of empathy, teamwork and integrated patient care [40]. However, these concepts have not been investigated together before in one study.

### Aim of this study and research question

In an attempt to clarify the concept of readiness, the current study uses a survey in all years of our medical school, which offered an opportunity to relate readiness for IPL to three well-known concepts in healthcare education curricula: professional identity, empathy and motivation. The research question is: How are professional identity, empathy and motivation associated with readiness for interprofessional learning in medical students? If these concepts help in clarifying ‘readiness for IPL’, they could also inform us about the embedding of IPL in the medical curriculum.

## Methods

### Design and setting

The present study is part of a large longitudinal study called the Student Motivation and Success study (SMS study) which includes collection of data from the students of our medical school for a number of sub-studies. During September 2015, all medical students from our school (3 years Bachelor and 3 years Master programme) were invited via electronic mail to participate in the SMS study [41]. Importantly, none of the medical students had experienced formal interprofessional teaching during their curriculum, meaning that our study was an opportunity to assess our students’ baseline readiness for IPL prior to them having any exposure to such teaching.

### Measures

The first part of the survey consisted of an informed consent and demographic information (gender, age, study year). In this current cross-sectional, questionnaire-based, exploratory study, the data collected by the use of 4 instruments was included.

**Professional Identity** was measured using a 10-item scale concerning affirmation and denial of identification with a particular group, tapping into three aspects of identity: awareness, affect and evaluation. A 5-point Likert scale was used in which 1 = ‘never’ and 5 = ‘very often’. The reliability of this scale has been reported with a Cronbach’s alpha value of 0.7, indicating an acceptable reliability [42].

We measured **empathy** with the 20-item Jefferson Scale of Empathy (student version: JSE-S), which has been validated in various studies on medical students [43]. Students self-reported their empathy on a scale of 1 (strongly disagree) to 7 (strongly agree). Hojat and LaNoue have reported a Cronbach’s alpha of 0.8 for the reliability of this measurement and an acceptable overall model fit for the three subscales [31]: Perspective Taking (10 items), Compassionate Care (8 items) and Walking in the patients’ shoes (2 items).

**Motivation for medical study** was measured using the SRQ-A: Self-Regulation Questionnaire-Academic [44] based on the Self-determination theory. The quality of motivation can be Controlled Motivation, which originates from external factors and ranges from external regulation to introjected regulation. Autonomous motivation (AM) originates from within an individual and ranges from identified regulation and integrated regulation to intrinsic regulation. The combination of AM and CM in individual students has been shown to be important in determining learning outcomes and student well-being [45]. More specifically a motivational profile with high AM and low CM is considered the most favourable in this regard [45]. The 16 items were rated on a 5-point Likert scale ranging from scale of 1 (strongly disagree) to 7 (strongly agree). Autonomous motivation was calculated using items for identified, integrated and intrinsic regulation and Controlled motivation was calculated using items for external and introjected regulation as described in the literature; Cronbach’s alphas above 0.7 have been reported for the reliability of AM and CM measurement, which are acceptable [45, 46].

To measure the **readiness for interprofessional learning**, the adapted version of the RIPLS [17] was used, with a 5-point Likert scale ranging from 1 (strongly disagree) to 5 (strongly agree). This version is used frequently [12, 16, 47, 48] and seemed the most appropriate version to use. Negative Professional Identity and Roles and Responsibilities subscales were reverse coded so that higher scores reflect a more positive attitude towards IPE [13]. The RIPLS measurement showed an acceptable internal consistency: the reported reliability was good for Teamwork & Collaboration, Positive Professional Identity, Negative Professional Identity, with Cronbach’s alphas of 0.80, 0.81, 0.76 respectively. The Cronbach’s alpha for Roles & Responsibilities was reported 0.40 [13, 17]. For an overview of all subscales and their number of items, see Table 1.

**Table 1:** Scales and subscales

Scale	Subscale	Subscale	Subscale	Subscale
Readiness for Interprofessional Learning Scale about willingness and ability for learning with, from and about other professions	Teamwork & Collaboration: shared learning will improve skills for T & C, communication, trust and ultimately patient care - TCIP	Positive Professional Identity: shared learning will benefit communication and teamwork skills and patient - PIP	Negative Professional Identity: shared learning is waste of time, learning clinical problem solving with own profession peers - NIP	Roles & Responsibilities: boundaries between profession of self and others, and hierarchies that may exist in clinical practice
Professional Identity Development: learning to function in professional context	Acknowledgement or denial of belonging to a professional group.			
Empathy: consisting of cognitive component PersTake and affective component with CompCare and WalkShoe.	Taking patient’s perspective into account when counselling - PersTake	Compassionate Care, showing concern for patient - CompCare	Walking in the patient’s shoes: try to place themselves in the position of the patient: – WalkShoe	
Motivation: valuing a cause and dedicating time and energy to it.	Controlled motivation: behaviour regulated by (expected) rewards or punishment from others. - CM		Autonomous motivation: behavior is guided by interest or valuing of activity. - AM	

### Statistical analysis

Statistical analysis was conducted using IBM SPSS Version 22. Pearson’s correlations were computed for all the variables (i.e. subscales) in the study. Dummy variables were defined for gender (female = 1, male = 0) and study year Bachelor B1 to B3, as well as Master M1 to M3. The internal consistencies of all subscales were computed. Linear regression was used to assess the association between readiness for interprofessional learning and Professional Identity, the subscales of Empathy (Perspective Taking, Compassionate Care and Walk in patient’s shoes) and Motivation (AM, CM) and to assess how good these measures can predict readiness for interprofessional learning. The difference between the observed and predicted values is used to assess the variance explained by the independent variables [49, 50].

RIPLS subscales Positive (PIP) and Negative Professional Identity (NIP) contain items regarding sharing expertise with students from different professions through team-based approaches to learning [10]. Therefore we report them as Shared Learning (SL) in the analysis, making it easier to distinguish these subscales from Brown’s Professional Identity measure. The RIPLS subscale Teamwork & Collaboration is shortened as TCIP. A hierarchical multiple regression approach was used to add Professional Identity, Empathy and Motivation in a second step (Table 2) to be able to analyse the explained variance associated with these variables additional to the variance explained by the demographic variables and study year.

**Table 2:** Depiction of the steps in the linear regression analysis

For TCIP	For SL
Step 1 (Model 1)	Step 1 (Model 1)
- Age	- Age
- Gender	- Gender
- Study Year	- Study Year
Step 2 (Model 2)	Step 2 (Model 2)
- Age	- Age
- Gender	- Gender
- Study Year	- Study Year
- Professional Identity	- Professional Identity
- Empathy: Perspective Taking, Compassionate Care, Walk in Patient’s Shoes	- Empathy: Perspective Taking, Compassionate Care, Walk in Patient’s Shoes
- Motivation: AM, CM	- Motivation: AM, CM

Gender(male = 0, female = 1) and Study year B1- M3 are defined as dummy variables

### Ethical considerations

Ethical approval was obtained from the Netherlands Association for Medical Education – Ethical Review Board (NVMO-ERB folder no. 388). Every participant gave written informed consent for participation and received a gift voucher for coffee or tea. We took measures to safeguard anonymity and confidentiality of all participants and report the research results only at a group level.

## Results

The survey was sent to 2451 medical students in Bachelor and Master and 947 students participated (response rate = 38%). The 10 Graduate Entry students (a special 4-year programme for students who already have a Biomedical/Health Sciences Bachelor) were excluded. Fifty-two students with missing values on all relevant variables were also excluded. See Table 3 Response rates of the participants and Table 4 Pearson correlations.

**Table 3:** Response rates of the participants (*n* = 885)

Year	B1	B2	B3	M1	M2	M3
Students	376	353	321	615 ^a^	499 ^a^	700 ^a^
n =	161	120	162	214	127	101
%	42.8	33.9	50.4	34.8	25.4	14.4

^a^ Due to the scheduling of clerkships, some students can be registered in two study years and therefore the numbers in the Master years are 1.5 or 2 times higher than the regular 350 students per year. For ‘n’ we used the study year that participants have indicated

**Table 4:** Pearson correlations

		Motivation		Readiness for Interprofessional Learning				Empathy		
	Age	AM	CM	NIP	PIP	SL	TCIP	Walk Shoe	Comp Care	Pers Take
Autonomous Motivation - AM	−0.162^**^									
Controlled Motivation - CM	0.054	−0.189^**^								
Negative Professional Identity - NIP	−0.080^*^	0.074^*^	− 0.084^*^							
Positive Professional Identity - PIP	−0.091^*^	0.156^**^	0.020	0.561^**^						
Shared Learning - SL	−0.093^*^	0.126^**^	− 0.040	0.902^**^	0.863^**^					
Teamwork & Collaboration - TCIP	−0.088^*^	0.178^**^	0.029	0.546^**^	0.708^**^	0.700^**^				
Walk in Patient’s Shoes - WalkShoe	−0.113^**^	− 0.085^*^	0.110^**^	− 0.115^**^	0.035	−0.046	0.019			
Compassionate Care - CompCare	−0.056	− 0.203^**^	0.148^**^	− 0.302^**^	− 0.161^**^	− 0.272^**^	− 0.243^**^	0.195^**^		
Perspective Taking - PersTake	−0.011	0.260^**^	− 0.117^**^	0.218^**^	0.242^**^	0.268^**^	0.338^**^	− 0.110^**^	− 0.555^**^	
Professional Identity	−0.167^**^	0.458^**^	− 0.136^**^	0.113^**^	0.056	0.092^*^	0.077^*^	− 0.087^*^	− 0.112^**^	0.158^**^

* Correlation is significant at the 0.05 level(2-tailed)

** Correlation is significant at the 0.01 level (2-tailed)

Cronbach’s alphas of all subscales except one were 0.7 or 0.8, indicating their good reliability (Table 5). The measurement of Roles & Responsibilities subscale of the RIPLS showed even lower internal consistency (Cronbach’s alpha 0.2) than in earlier studies [13, 17], therefore, this was discarded in the linear regression.

**Table 5:** Descriptive Statistics

Variables [n = items in scale] Range of scores	Mean	Std. Deviation	N	Cronbach’s Alpha
Age	22	3.17	885	–
Gender	25% male		885	–
Professional Identity [10] Range: 10–50	3.88	0.51	776	0.8
Empathy - Walk in Patient’s Shoes [2] Range: 2–14	3.54	1.19	676	0.7
Empathy - Compassionate Care [8] Range: 8–56	2.46	0.74	663	0.7
Empathy - Take Patient’s perspective [10] Range: 10–70	5.56	0.66	660	0.8
Motivation - Autonomous - AM [8] Range: 8–40	4,26	0.51	831	0.8
Motivation - Controlled - CM [8] Range: 8–40	1.90	0.69	834	0.8
RIPLS - Shared Learning - NIP+PIP [3 + 4] Range: 7–35	7.07	1.32	732	Composed of NIP + PIP
RIPLS - Negative Profess. Identity - NIP [3] Range: 3–15	3.57	0.81	742	0.7
RIPLS - Positive Profess. Identity - PIP [4] Range: 4–20	3.50	0.67	736	0.8
RIPLS – Teamwork & Collaboration - TCIP [9] Range: 9–45	3.84	0.47	734	0.8
RIPLS - Roles & Responsibilities [3] Range: 3–15	9.57	1.78	744	0.2

A two-step **linear regression** analysis was performed separately for the RIPLS subscales Teamwork & Collaboration (TCIP) and Shared Learning (SL, comprised of NIP and PIP) as dependent variables. The scores per study year were relative to the last study year (M3).

### Teamwork & Collaboration (TCIP)

In **Step 1** resulting in Model 1, age, gender and study year were entered, which explained 3.6% of the variance in TCIP. Being female and B1 study year seems to predict a higher TCIP score (*p* <  0.05).

**Step 2 for TCIP** - After entry of the Professional Identity, Empathy subscales and Motivation subscales in Step 2, the total variance in Teamwork & Collaboration explained by the model as a whole was 15.7%, *p* <  0.01. The measurements in model 2 explained an additional 12.1% of the variance in readiness, F change =14.866, *p* < 0.005. Perspective Taking, Autonomous and Controlled motivation seems to predict Teamwork & Collaboration measured by this subscale of RIPLS. Compassionate care seems to predict lower Teamwork & Collaboration. Being female and study year B1 have a significant contribution in explaining TCIP in model 1, but not in model 2 (Table 6).

**Table 6:** Model TCIP

Model Teamwork & Collaboration (RIPLS subscale TCIP)		Unstandardized Coefficients	*p*-value	95,0% Confidence Interval for B	
		B		Lower Bound	Upper Bound
1	(Constant)	3.692	< 0.001	3.220	4.164
	Age Gender (male = 0; female = 1)	−0.004 0.094*	0.663 0.028	−.0021 0.010	0.013 0.178
	Study year B1 Study year B2 Study year B3 Study year M1 Study year M2	0.211* 0.000 0.018 0.074 0.079	0.020 0.996 0.815 0.285 .0282	0.033 −0.176 − 0.135 − 0.061 − 0.065	0.388 0.177 0.171 0.209 0.224
2	(Constant)	2.788	< 0.001	2.054	3.522
	Age	−0.009	0.294	−0.025	0.008
	Gender (male = 0; female = 1)	0.060	0.144	−0.020	0.139
	Study year B1 Study year B2 Study year B3 Study year M1 Study year M2 Perspective Taking Compassionate Care Walk in patient’s shoes Professional Identity Autonomous motivation - AM Controlled motivation - CM	0.125 −0.064 − 0.068 − 0.032 0.057 0.170* − 0.092* 0.024 − 0.043 0.085* 0.076*	0.150 0.459 0.358 0.634 0.409 < 0.001 0.004 0.119 0.272 0.047 0.004	−0.045 − 0.232 − 0.214 − 0.162 − 0.079 0.103 − 0.153 − 0.006 − 0.120 0.001 0.024	0.296 0.105 0.077 0.098 0.194 0.236 − 0.030 0.054 0.034 0.168 0.128

Study years were coded as dummies; study years B1 to B3 and M1 to M2 were compared to M3

### Shared learning (SL)

In **Step 1** resulting in Model 1, age, gender and study year were entered, which explained 4.3% of the variance in SL. Being female and in study year B1 seems to predict higher score for shared learning, *p* < 0.05.

**Step 2 for SL** - After entry of the aforementioned subscales, the total variance in Shared Learning explained by the model was 14.3%. The measurements added in model 2 explained an additional 10.0% of the variance in readiness, F change = 12.049, p = < 0.0005. Being female, in study year B1 and ‘Perspective Taking’ seem to predict shared learning (p < 0.05), ‘Compassionate care’ seems to predict lower shared learning. This indicates that the ability to show more compassionate care diminishes the students’ readiness for Shared Learning. Apparently, motivation has a less prominent association with Shared Learning (Table 7).

**Table 7:** Model SL

Model Shared Learning (RIPLS subscales Neg and Pos Professional Identity)		Unstandardized Coefficients	*p*-value	95,0% Confidence Interval for B	
		B		Lower Bound	Upper Bound
1	(Constant) Age Gender (male = 0; female = 1)	6.319 −0.004 0.390*	< 0.001 0.883 0.001	5.002 − 0.051 0.156	7.636 0.044 0.624
	Study year B1 Study year B2 Study year B3 Study year M1 Study year M2	0.549* 0.133 − 0.057 0.116 0.040	0.030 0.595 0.794 0.547 0.845	0.054 − 0.358 − 0.483 − 0.261 − 0.364	1.043 0.625 0.369 0.493 0.444
2	(Constant) Age Gender (male = 0; female = 1)	6.481 −0.012 0.281	< 0.001 0.608 0.015	4.408 −0.058 0.056	8.554 0.034 0.507
	Study year B1 Study year B2 Study year B3 Study year M1 Study year M2 Perspective taking Compassionate Care Walk in Patient’s Shoes Professional Identity Autonomous Motivation (AM) Controlled Motivation (CM)	0.501 0.153 − 0.154 − 0.041 0.055 0.226* − 0.463* 0.000 0.021 − 0.021 0.084	0.042 0.529 0.464 0.827 0.779 0.018 < 0.001 0.997 0.848 0.862 0.260	0.019 − 0.324 − 0.565 − 0.408 − 0.331 0.039 − 0.637 − 0.085 − 0.196 − 0.257 − 0,062	0.982 0.629 0.258 0.326 0.441 0.412 − 0.290 0.085 0.238 0.215 0,231

Study years were coded as dummies; study years B1 to B3 and M1 to M2 were compared to M3

Thus, gender (being female) has an association with Shared Learning. Autonomous and controlled motivation have an association with Teamwork & Collaboration, whereas motivation was not significantly associated with Shared Learning. Professional Identity was not significant in explaining Teamwork & Collaboration and Shared Learning. Two subscales of empathy were associated with both Teamwork & Collaboration and Shared Learning and therefore seem to play an important role in readiness for IPL.

## Discussion

In an attempt to clarify the concept of readiness for interprofessional learning, we intended to find variables explaining the variance in readiness for IPL, using elements from the medical curriculum that have been measured extensively: professional identity development, empathy and motivation for medical school. These elements also feature in readiness for learning in general, more specifically ‘ownership of learning’ [21].

### Professional identity

We cannot conclude that students with stronger developed professional identity show less readiness for IPL. The RIPLS subscale Professional Identity, comprising of Negative and Positive Professional identity, appeared to measure different concepts than the Professional identity scale we used [42]. We agree with Williams, Brown and Boyle (2012) to regard this subscale as concerning Shared Learning [14].

### Empathy

RIPLS Teamwork & Collaboration and Shared Learning were associated with ‘Perspective Taking’ and ‘Compassionate Care‘ from the Empathy scale, but not with ‘Walking in Patient’s shoes’. The association of Compassionate care with Teamwork & Collaboration and Shared Learning was negative, which could be due to the phrasing. Examples of items in the subscale Compassionate Care are: ‘I believe that emotion has no place in the treatment of medical illness.’ and ‘Attentiveness to patients’ personal experiences does not influence treatment outcomes.’ The wording of these items make compassionate care an individual construct, therefore we were not surprised that it was not associated with teamwork or shared learning.

Our study confirms previous findings that empathy plays a role in teamwork. Hojat et al. (2014) found a significant overlap between empathy, teamwork and integrative patient care, applying the Jefferson scales of Empathy for students (JSE-S) and two scales measuring collaboration and orientation toward an integrative approach to patient care [40]. They suggest that implementation of such integrative care in the medical education curriculum may lead to improved empathic engagement in patient care and more positive orientation toward teamwork and collaborative care. In our study, the explanatory role of ‘perspective taking’ prevails and is positive. As we hypothesized, a higher score on this cognitive component of empathy is associated with higher readiness for IPL. Incorporating perspective taking with IPL/IPC has been done in two instruments, namely ICCAS and IPAS [49, 50]. Examples of such items in the ICCAS are:

*Item 15. Include the patient/family in decision-making;*

*Item 16. Actively listen to the perspectives of IP team members;*

*Item 17. Take into account the ideas of IP team members.*

Examples of such items in the IPAS are:

*Item: ‘It is important for me to understand the patient’s side of the problem’ and Item: ‘It is important for me to communicate compassion to my patients‘.*

Our study suggests that in the medical curriculum, the cognitive component of empathy i.e. perspective taking, might need to precede the introduction of IPL/IPC.

### Motivation

Autonomous and Controlled motivation seemed to predict Teamwork & Collaboration, but not Shared Learning. The lack of association between shared learning and motivation could be due to the motivation items being specifically regarding the medical study, and not IPL.

Because our participants had not experienced IPL in their curriculum, they were not familiar with shared learning with students from other professions. As for the explanatory effect of study year B1 in the first step: we measured in September, when fresh students just entered medical school and their answers could be idealistic rather than reflecting their experience with professional roles, teamwork or shared learning.

The RIPLS subscale Roles & Responsibilities did not show a reliable measurement in our study. From its development, the RIPLS has lacked a theoretical framework [18] and this subscale has been low in reliability [17]. Perhaps the instructions for the use of the scale were not clear and users of the RIPLS have not reverse coded the items with a negative wording [51]. Schmitz and Brandt (2015) argued that communication, collaboration, and teamwork are closely linked and that it is difficult to evade high inter-correlations [52].

Using the RIPLS as a pre–test when students have not experienced the IPE intervention, could measure an experienced deficit of ownership of learning as students may lack goal orientation or self-direction regarding IPL. We therefore consider the RIPLS as a suboptimal instrument, which does not clarify the ‘what’ and ‘how’ of IPL in a curriculum.

### Implications of the study

Measurement of Readiness for IPL seems to have a limited advisory role in the implementation of IPL. A pre-post comparison of readiness for IPL as an outcome measure of IPL also seems to be of limited value, especially when accreditation bodies demand measurement of outcomes at higher Kirkpatrick’s levels.

#### Limitations

The present study was conducted at one medical school, which is a limitation for the transferability of findings. The low internal consistency of the Roles and Responsibility subscale was another limitation. We recommend development of a new subscale for measuring this concept.

## Conclusions

‘Readiness for IPL’ should not be considered as a threshold that students pass and then learn about IPL. The ability and willingness to take the perspective of someone else is an essential element of empathy and also seems to be essential in readiness for interprofessional learning. ‘The ‘what’ of IPL and ‘how it can be placed in the curriculum’ may benefit from a combination with the cognitive component of empathy (‘Perspective taking’) and elements in the curriculum that promote autonomous motivation, so that students gradually develop ownership of *interprofessional* learning.

## Abbreviations

AM: Autonomous Motivation
B 1–3: Bachelor study year 1 to 3
CM: Controlled Motivation
ICCAS: Interprofessional Collaboration Competency Attainment Scale
IPAS: Interprofessional Attitudes Scale
IPC: Interprofessional Collaboration
IPE: Interprofessional Education
IPL: Interprofessional Learning
M 1–3: Master study year 1–3
RIPLS: Readiness for Interprofessional Learning Scale
SDT: Self-determination theory of motivation
SL: Shared learning (subscale composed of the Positive and Negative Professional Identity subscales of RIPLS)
SRQ-A: Self-Regulation Questionnaire-Academic
TCIP: Teamwork and Collaboration (subscale of RIPLS)
